# Cheerfulness

**Published:** 1896-04

**Authors:** 


### Cheerfulness.

Cheerfulness, first of all, is a duty a man owes to himself. Any physician will tell you that one of the best preventives of disease is cheerfulness, and one of the best curatives when disease has set in, is that happy and hopeful disposition that feels it is all for the best, whatever comes, health or sickness, life or what they call death. Such a man will be restored to health under the most adverse circumstances, while the strong but despondent and melancholy disposition will weaken and fade away. All the doctors in the land cannot save a man who has not energy enough to will his own existence. Thinking of this, the great English historian said that cheerfulness was worth more than five thousand pounds a year.

Moreover, a man owes it to his family to cast trouble away when he comes to his home. He is a selfish being who, having trouble in his trade or calling or profession, always brings his trouble home for the purpose of making every one else miserable. There is no bravery or manliness in that kind of thing, no thoughtfulness for others' happiness, no trust in God that all will be for the best. The one at home may also forget and worry the already tired man with complaints beyond his power to change. It may, therefore, be set down as an axiom that home happiness depends upon the woman meeting the man with a pleasant face, though her body may be weary and her heart sick; it depends upon the man having a kind and a cheerful countenance, though that day all had gone wrong, and he had found, or thought he had found, that all men were liars.

It comes naturally to pass that if cheerfulness is a duty a man owes to himself and his family, it is also one he owes to the nation and age in which he lives. What are commonly called hard times are caused, in nine cases out of ten, by the people, as a nation, slowly or suddenly getting despondent, slowly or suddenly failing to trust in the nation as a nation. But in reality there is usually no reason for hard times. There is just as much money in America, and more, than there was a few years ago. The crops have not failed, nor the honesty of trade, more than at any other time. Think of the humble way our fathers lived. The people simply fall into the bad habit of saying times are hard and money tight. After a while so much talk leads men to believe something is wrong, and then every man fails to work as hard as he can, holding every cent of money he can get hold of, and thereby comes hard times. It is like the sweep of a pestilence over a land. More than half the people who get the cholera, or any other scourge, get it by fear. Every man who joins in the talk about hard times will suffer from what his own words and want of trust may bring. After a while, when men find that it yields rather poor interest keeping their capital locked up, they will begin again to send their money out, and then, in commercial language "confidence will be restored." After awhile, when men get to believing in God, and to giving his church as they ought to give his divine body, God will open the windows of heaven, as of old, and the blessings of better times will come.—*Dietetic and Hygienic Gazette.*

